# Supplementary material for: Low-THz Vibrations of Biological Membranes
Source: Membranes (Basel). 2023 Jan 21;13(2):139. doi: 10.3390/membranes13020139 (PMC9965665; doi:10.3390/membranes13020139)
Supplement: Supplementary file 1 [file membranes-13-00139-s001.zip › membranes-2116721-supplementary.pdf]

Article

Supplementary Materials: Low-THz Vibrations of Biological Membranes

Chloe Luyet [1], Paolo Elvati [2], Jordan Vinh [3] and Angela Violi [1,2,4,*]

[1] Chemical Engineering, University of Michigan, Ann Arbor, MI, 48109-2125, USA
[2] Mechanical Engineering, University of Michigan, Ann Arbor, MI, 48109-2125, USA
[3] Biomedical Engineering, University of Michigan, Ann Arbor, MI, 48109-2125, USA
[4] Electrical Engineering and Computer Science, University of Michigan, Ann Arbor, MI, 48109-2125, USA
* Correspondence: avioli@umich.edu

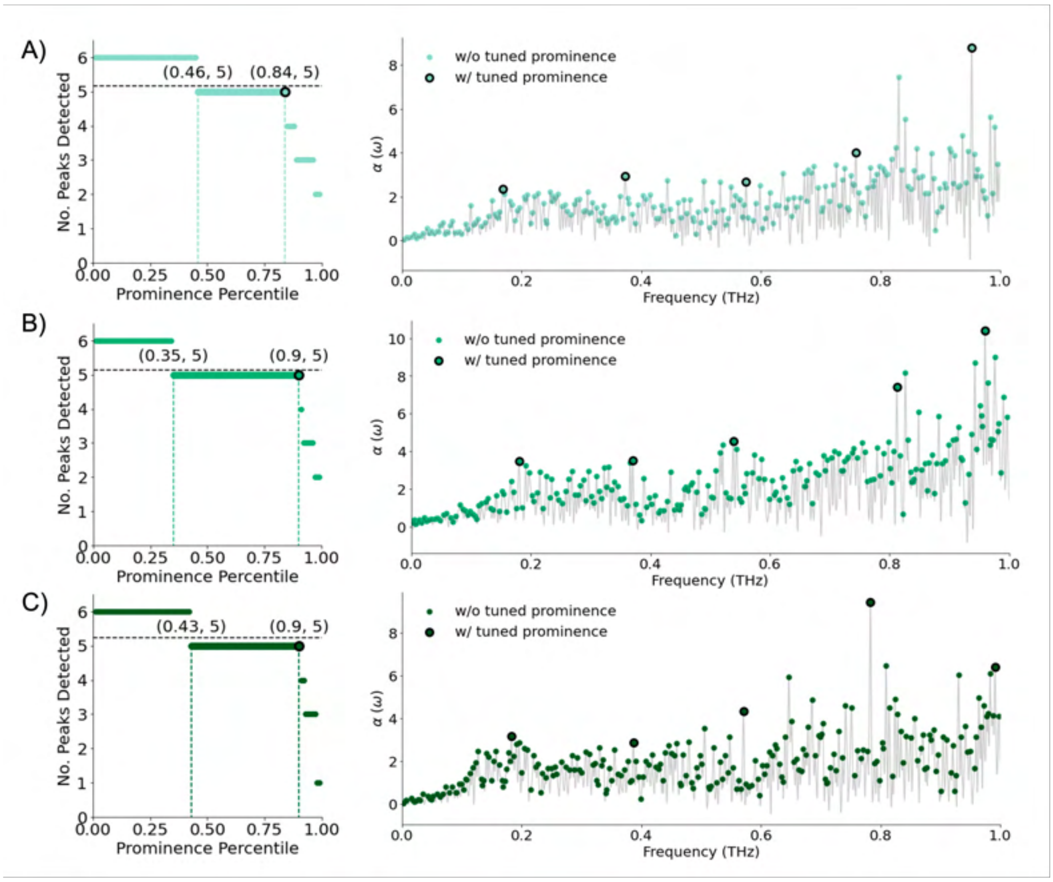

Figure S1. Prominence Filter Tuning. Demonstration of prominence filter tuning in signal detection for 3 replicas of the $S476^{A}_{33}$ membrane. Each panel represents a different replica. Final peaks are determined by taking the average and standard error of the mean for each tuned peak across replicas. (left) Step function plots show the number of peaks as a function of the prominence threshold, with the dashed line indicating the average number of peaks; the black circle shows the final threshold value and number in parentheses demarcate the percentile interval of thresholds that detect a number of peaks close to the average.

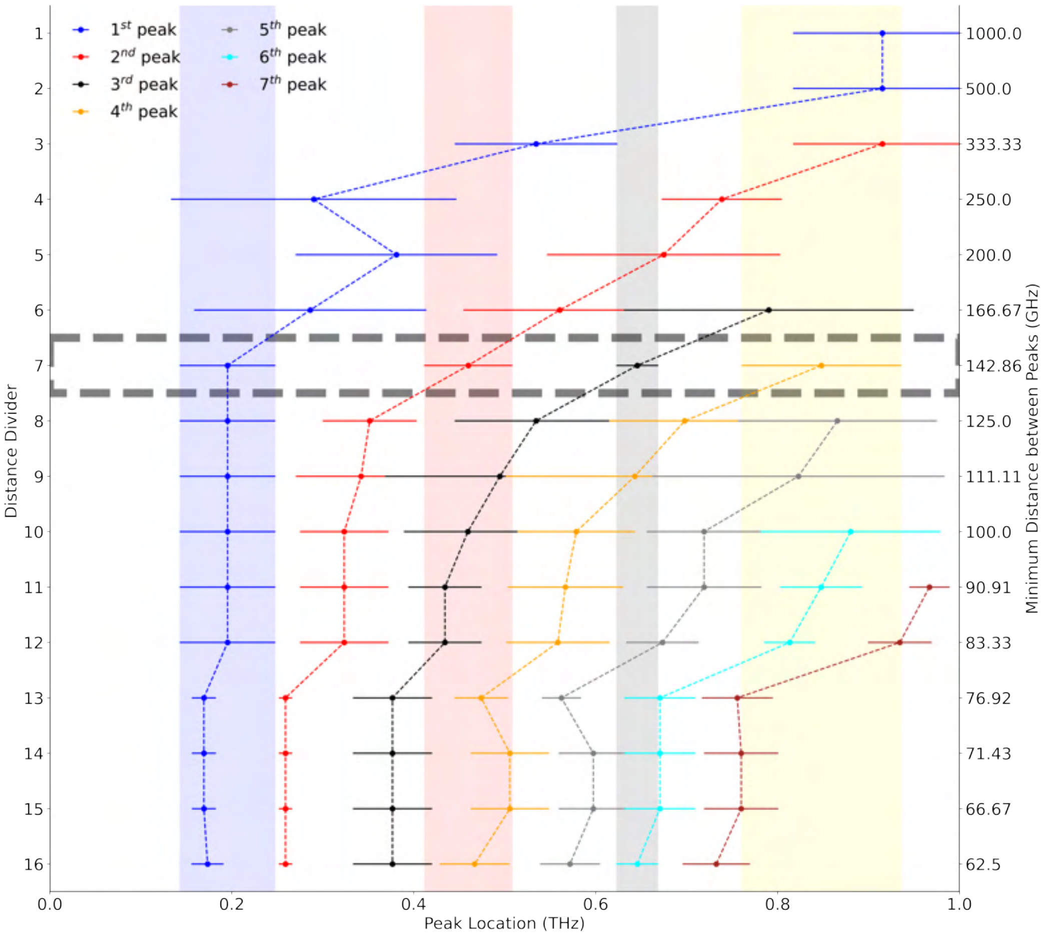

Figure S2. Distance Filter Selection for $S476^A_{51}$. Horizontal, gray, dotted box highlights the distance filter chosen for $S476^A_{51}$ membranes. Shaded blue (first peak), red (second peak), gray (third peak), and yellow (fourth peak) demonstrate the region of convergence of spectra peaks.

Table S1. Asymmetric *S. aureus* Membrane Compositions per Leaflet. Asymmetric *S. aureus* Membrane Compositions for $S476^A_{51}$ and $S476^A_{33}$. The subscript represents the total concentration of LPG in the membrane, as determined by Rehal *et al.*

**Leaflet**	PG	LPG	CL
**Upper**	10%	95%	20%
**Lower**	90%	5%	80%

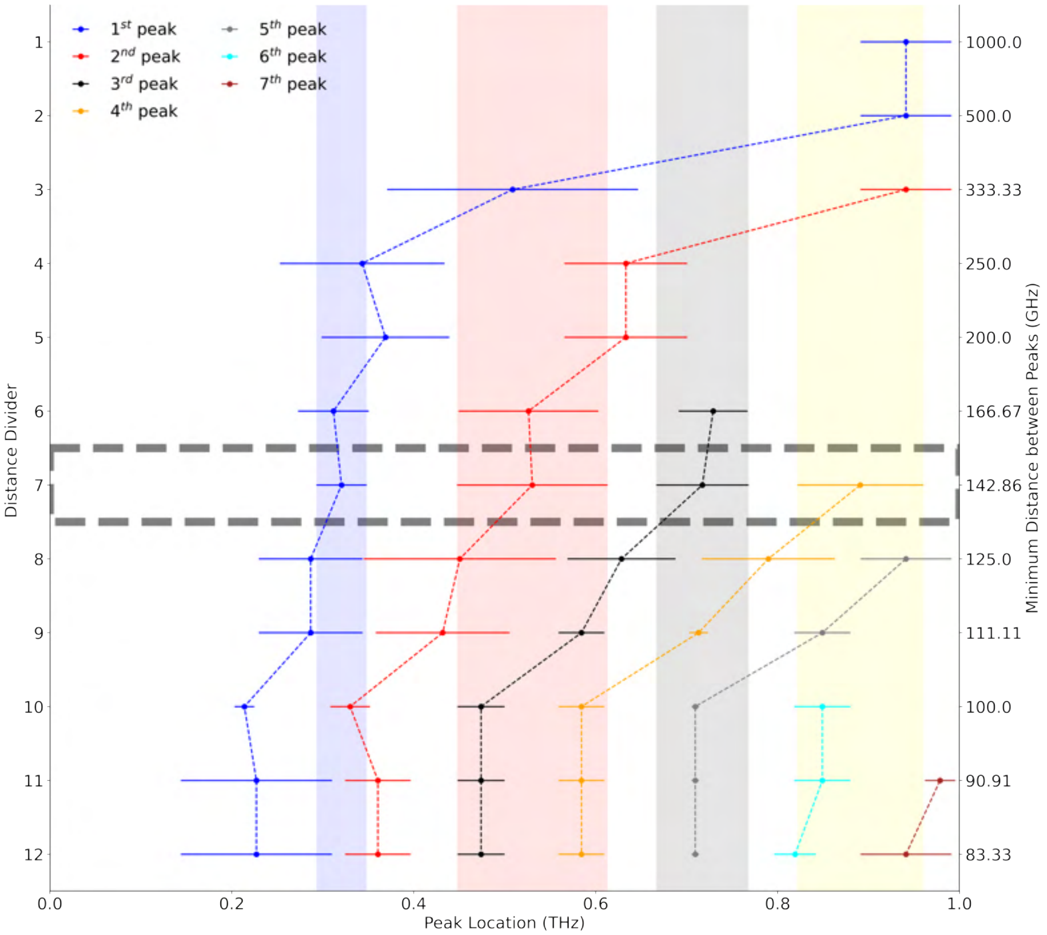

Figure S3. Distance Filter Selection for *B. Subtilis* Membrane. Horizontal, gray, dotted box highlights the distance filter chosen for *B. Subtilis* membrane. Shaded blue (first peak), red (second peak), gray (third peak), and yellow (fourth peak) demonstrate the region of convergence of spectra peaks.

Table S2. Detailed Membrane Compositions. Detailed membrane compositions by lipid types. For *S. aureus* membranes, PG fatty acid tail is POPG, LPG fatty acid tail is PLPG, and CL is TMCL1. For rat liver plasma membranes, CL is PVCL2, PE is POPE, PC is POPC, PI is POPI, and PS is POPS.

**Type**	Leaflet	PG	LPG	CL	FA	DAG	PE	PC	PI	PS	PSM	CHL
$S476^{A}_{51}$	Upper	48.5%	4.3%	1.2%	-	-	-	-	-	-	-	-
	Lower	2.5%	38.7%	4.8%	-	-	-	-	-	-	-	-
$S476^{A}_{33}$	Upper	32.3%	6.2%	0.8%	-	-	-	-	-	-	-	-
	Lower	1.7%	55.8%	3.2%	-	-	-	-	-	-	-	-
$S476_{33}$	Both	62%	34	4%	-	-	-	-	-	-	-	-
B. subtilis	Both	9.5%	-	2.9%	1.9%	30.2%	55.5%	-	-	-	-	-
**Rat Liver**	Both	-	-	1%	-	-	15%	25%	5%	6%	11%	37%

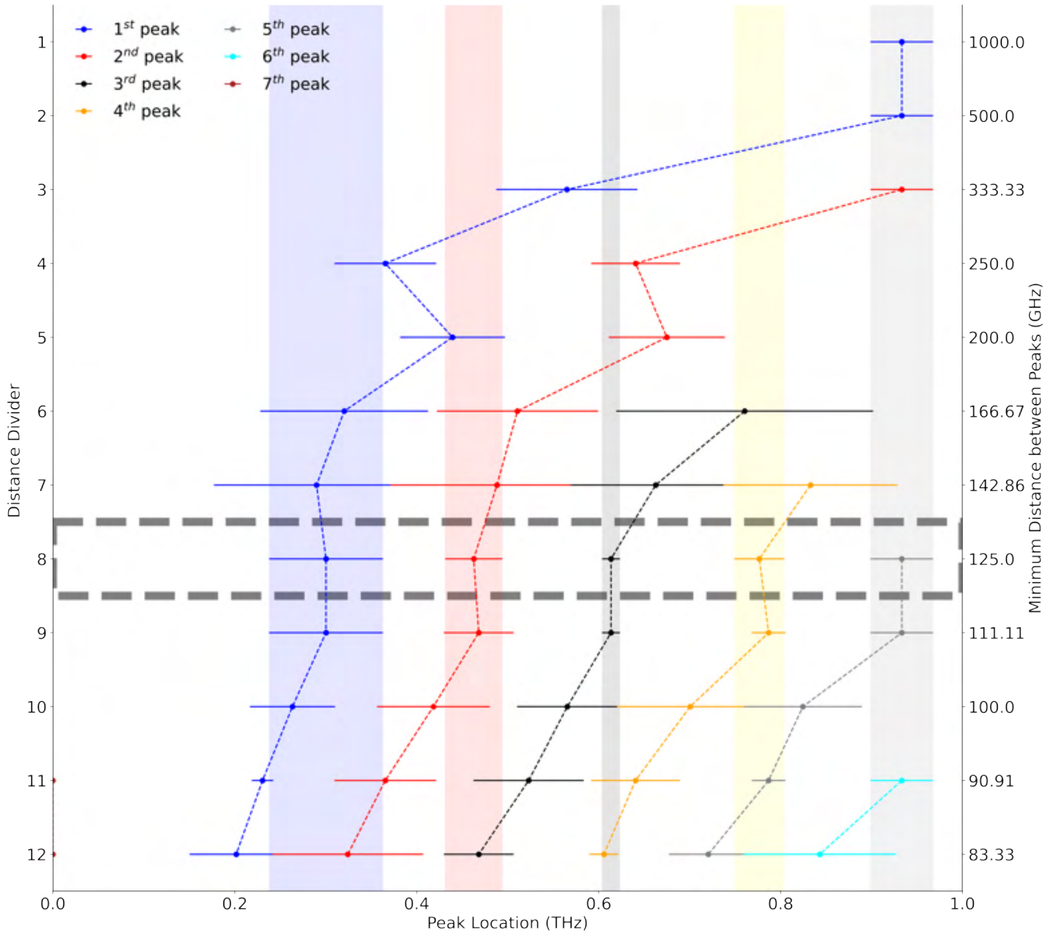

Figure S4. Distance Filter Selection for Rat Liver Plasma Membrane. Horizontal, gray, dotted box highlights the distance filter chosen for rat liver plasma membrane. Shaded blue (first peak), red (second peak), gray (third peak), yellow (fourth peak), and light gray (fifth peak) demonstrate the region of convergence of spectra peaks.

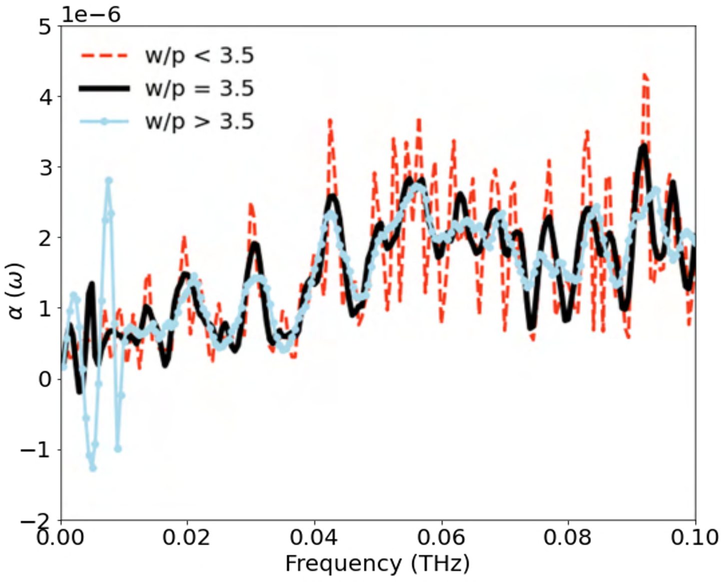

Figure S5. Savitsky-Golay Filter Selection. Savitsky-Golay Parameter Selection Demo shows why we chose a ratio between window and polynomial order of 3.5 (solid, black line) to filter our data. Data in this figure is not normalized.

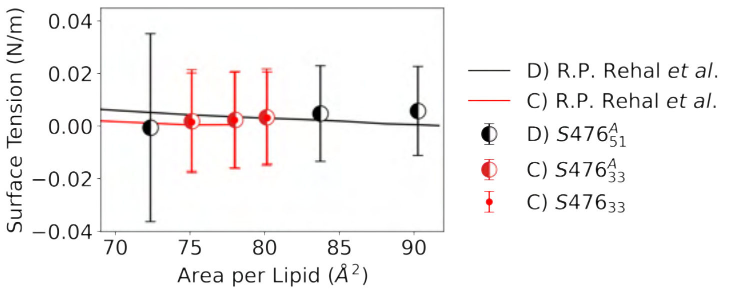

Figure S6. Langmuir Isotherms. Langmuir Isotherms for *S. aureus* membranes studied in this paper and in Rehal *et al.* Bars indicate standard error of the mean.

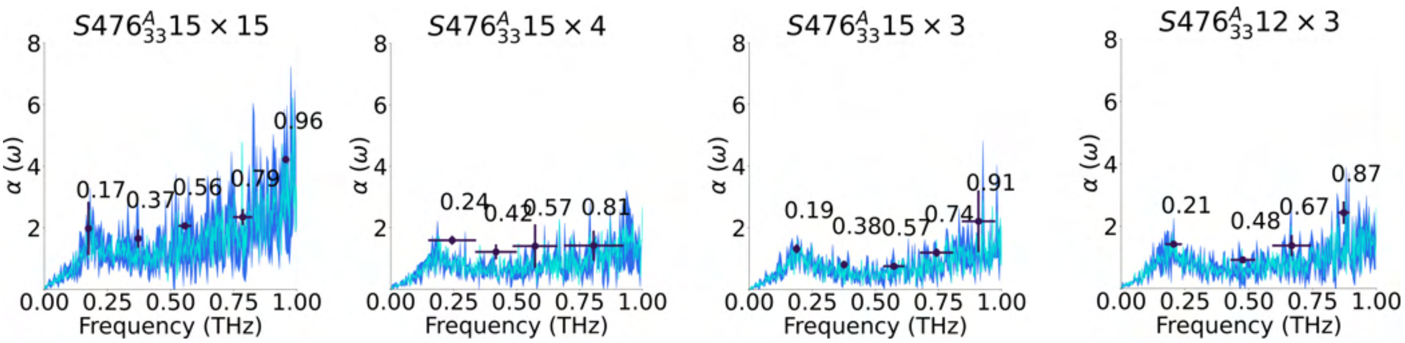

Figure S7. Effect of $S476^A_{33}$ Periodic Boundary Size on Spectra after Normalization. Normalization occurs after the filter is applied. Average spectra is in cyan, and standard deviation among replicas for spectra is in dark blue. Bars indicate standard deviation for peaks.

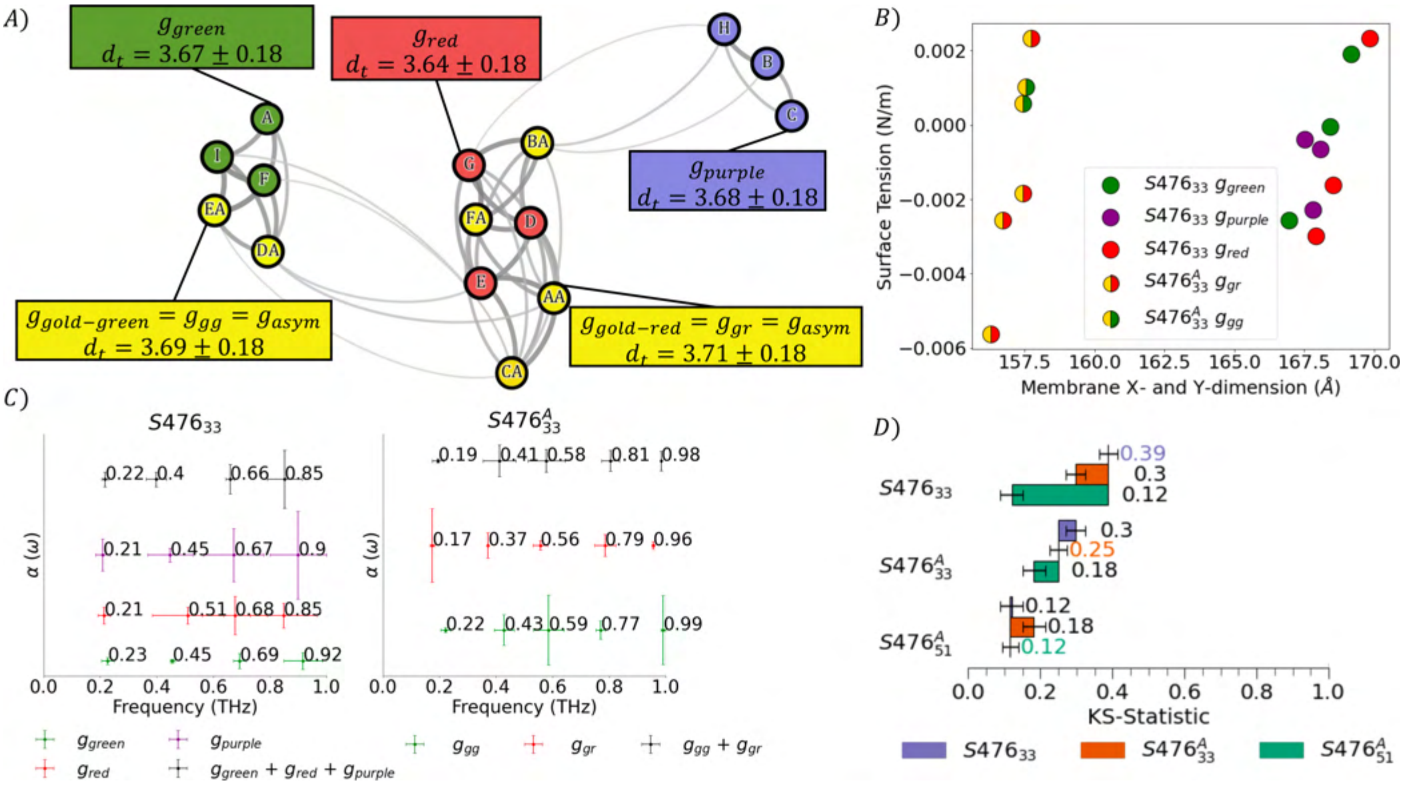

Figure S8. Clustering Analysis for KS Statistics of $S476_{33}$ and $S476^A_{33}$ Replicas. (A) Fully connected weighted graph created using (1 - KS-statistics) as the weight for each edge. Nodes represent replicas of S476$^A_{33}$ and S476$_{33}$; only edges with weight higher than 0.75 are shown. Colors indicate modularity clustering; $S476_{33}$ spectra are grouped into 3 clusters (g_{green}, g_{red}, and g_{purple},) and the $S476^A_{33}$ spectra into 2 groups (g_{gg} and g_{gr}). Groups g_{red} and g_{gr} were chosen in the main text. Membrane thickness (d_t) with its standard deviation is reported for each group/membrane type. (B) Membrane dimension and surface tension of the system are also not determinants of grouping. Errors are not shown, as standard deviations are insignificant compared to marker size. (C) Both peak location and peak intensity among replicas in different groups; peak locations are staggered upward on the relative absorption scale for clarity. Error bars indicate standard deviation. (D) KS-statistics comparison among *S. aureus* membranes as a result of averaging groups (g_{green}, g_{red}, and g_{purple} to make $S476_{33}$, and groups g_{gg} and g_{gr} to make $S476^A_{33}$) shows that the spectra from symmetric and asymmetric membranes are still statistically indistinguishable. Error bars represent the standard error of the mean.

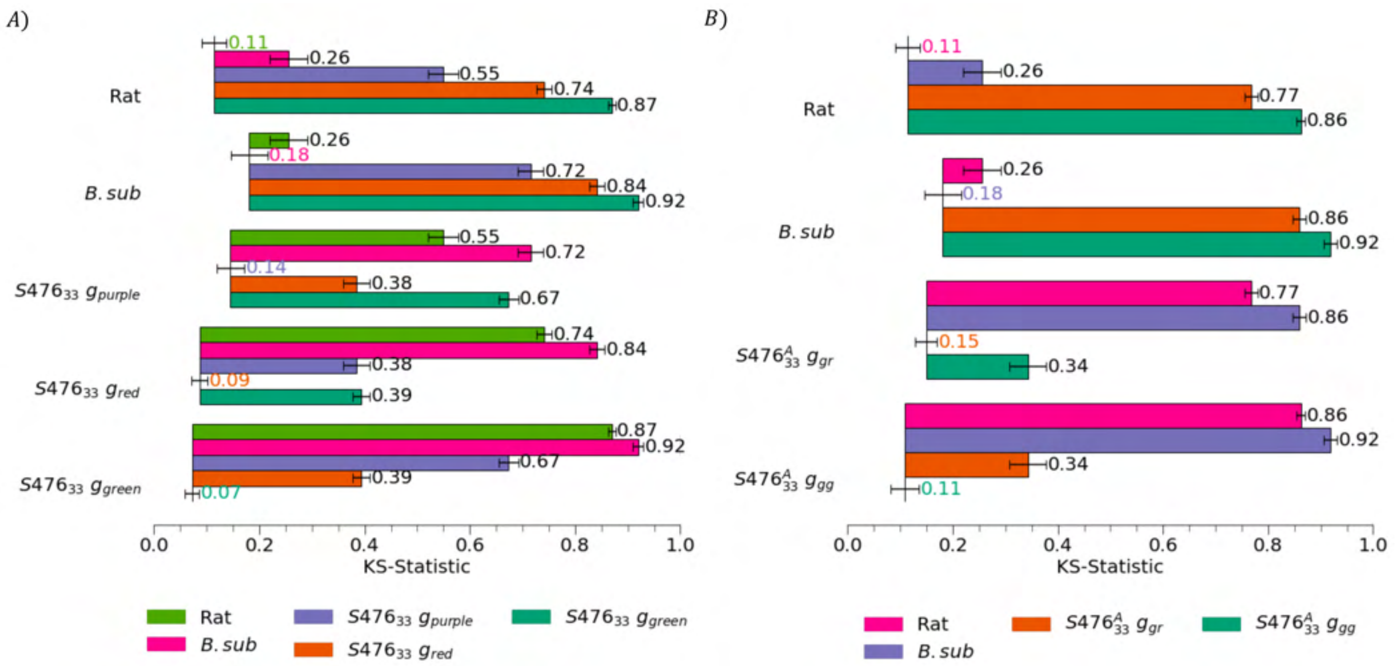

Figure S9. KS Statistics for S476$_{33}$ and S476$^{A}_{33}$ Clusters. KS statistics for each S476$_{33}$ (A) and S476$^{A}_{33}$ (B) cluster (see Fig. S8A for definition), shows that independently of the chosen group, the spectra of *S. aureus*, *B. Subtilis* and rat liver cell are distinguishable. Error bars represent the standard error of the mean.

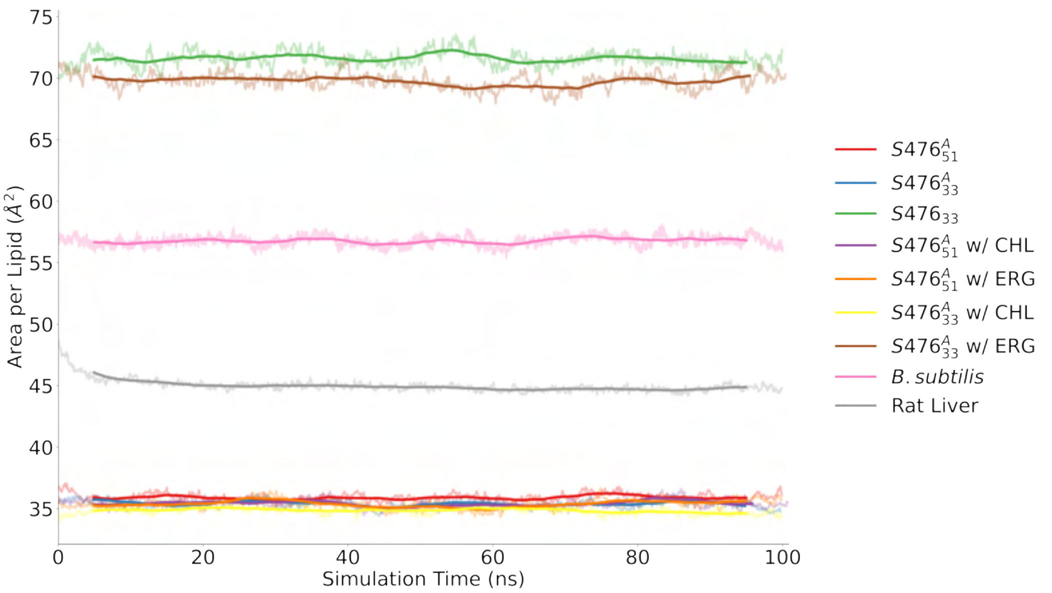

Figure S10. Area per Lipid Equilibration. Area per Lipid over Time from NPsT Ensemble shows converged and stable membrane systems. Faint lines are raw area per lipids calculated over time, and solid lines are the moving averages of area per lipid for each membrane.

Table S3. Complete KS Statistics. KS Statistics, where KS statistic is reported to three significant figures. Errors are standard errors of the mean. $\otimes$ indicates that the membrane does not contain sterols, CHL indicates the membrane contains cholesterol, and ERG indicates the membrane contains ergosterol.

**Type**	KS Statistic
**S476$_{51}^{A}$ ($\otimes$) vs. S476$_{51}^{A}$ ($\otimes$)**	0.12 ± 0.02
**S476$_{51}^{A}$ ($\otimes$) vs. S476$_{33}^{A}$ ($\otimes$)**	0.12 ± 0.02
**S476$_{51}^{A}$ ($\otimes$) vs. S476$_{33}$ ($\otimes$)**	0.08 ± 0.01
**S476$_{51}^{A}$ ($\otimes$) vs. S476$_{51}^{A}$ (CHL)**	0.21 ± 0.05
**S476$_{51}^{A}$ ($\otimes$) vs. S476$_{51}^{A}$ (ERG)**	0.12 ± 0.02
**S476$_{33}^{A}$ ($\otimes$) vs. S476$_{33}^{A}$ ($\otimes$)**	0.15 ± 0.02
**S476$_{33}^{A}$ ($\otimes$) vs. S476$_{33}$ ($\otimes$)**	0.11 ± 0.01
**S476$_{33}^{A}$ ($\otimes$) vs. S476$_{33}^{A}$ (CHL)**	0.33 ± 0.04
**S476$_{33}^{A}$ ($\otimes$) vs. S476$_{33}^{A}$ (ERG)**	0.23 ± 0.03
**S476$_{33}^{A}$ ($\otimes$) vs. *B. Subtilis***	0.86 ± 0.01
**S476$_{33}^{A}$ ($\otimes$) vs. Rat Liver**	0.77 ± 0.01
**S476$_{33}$ ($\otimes$) vs. S476$_{33}$ ($\otimes$)**	0.09 ± 0.02
**S476$_{51}^{A}$ (CHL) vs. S476$_{51}^{A}$ (CHL)**	0.22 ± 0.05
**S476$_{51}^{A}$ (CHL) vs. S476$_{51}^{A}$ (ERG)**	0.17 ± 0.04
**S476$_{33}^{A}$ (CHL) vs. S476$_{33}^{A}$ (CHL)**	0.19 ± 0.04
**S476$_{33}^{A}$ (CHL) vs. S476$_{33}^{A}$ (ERG)**	0.45 ± 0.05
**S476$_{33}^{A}$ (ERG) vs. S476$_{33}^{A}$ (ERG)**	0.15 ± 0.03
**S476$_{33}^{A}$ (ERG) vs. S476$_{33}^{A}$ (ERG)**	0.25 ± 0.06
B. Subtilis* vs. *B. Subtilis	0.18 ± 0.03
***B. Subtilis* vs. Rat Liver**	0.30 ± 0.04
**Rat Liver vs. Rat Liver**	0.11 ± 0.02

```
// Dipole Calculation TCL Script
// Step 1 in Figure 2
#!/usr/bin/tclsh

set name1 "psf_file"
set name2 "pdb_file"
set selection "segid MEMB"

set ipsf ${name1}.psf
set idcd ${name2}.dcd

mol load psf $ipsf dcd $idcd

set sel [atomselect top ${selection}]

set totq [vecsum [join [${sel} get charge] { }]]
vmdcon -info "Selection net charge: $totq"

set of [open ${name2}.dipole.txt w]
puts $of [format "# D_tot Dx  Dy    Dz"]
set numframes [molinfo top get numframes]

for {set i 0} { $i < ${numframes} } {incr i} {
  ${sel} frame $i
  set dipv [measure dipole $sel -masscenter]
  set Dx [lindex ${dipv} 0]
  set Dy [lindex ${dipv} 1]
  set Dz [lindex ${dipv} 2]
  set D [expr sqrt(${Dx}**2 + ${Dy}**2 + ${Dz}**2) ]
  puts ${of} [format "%6g %6g %6g %6g" $D ${Dx} ${Dy} ${Dz}]
  flush ${of}
}

close ${of}
exit
```
